# Disseminated zoster with vasculitis

**DOI:** 10.1016/j.jdcr.2022.08.002

**Published:** 2022-08-10

**Authors:** Raymond Zhao, Daniel A. Nadelman, Paul W. Harms, Milad Eshaq

**Affiliations:** a Department of Dermatology, University of Michigan Medical School, Ann Arbor, Michigan; b Department of Pathology, University of Michigan Medical School, Ann Arbor, Michigan

**Keywords:** herpes virus, leukocytoclastic vasculitis, small-vessel vasculitis, varicella-zoster virus, LCV, leukocytoclastic vasculitis, VZV, varicella-zoster virus

## Introduction

Varicella-zoster virus (VZV) is a human herpes virus responsible for varicella (chickenpox) and zoster (shingles). During primary infection, virions establish latency in dorsal root ganglia, which can later reactivate and present with the characteristic dermatomal vesicular rash of zoster.^1^ Immunocompromised and elderly individuals are particularly at risk of reactivation due to relative suppression of their cell-mediated immunity.^1^ In addition to the well-known complications of zoster paresis and postherpetic neuralgia, VZV can also establish infection in the arterial wall, leading to inflammatory vasculopathies varying from discrete capillaritis to granulomatous vasculitis and obliterative angiitis.^2^ However, cutaneous vasculitic presentations due to viropathic changes are rare, and less than a dozen cases have been reported so far. Here, we report a case of disseminated zoster with a component of vasculitis.

## Case report

**Fig 1:**
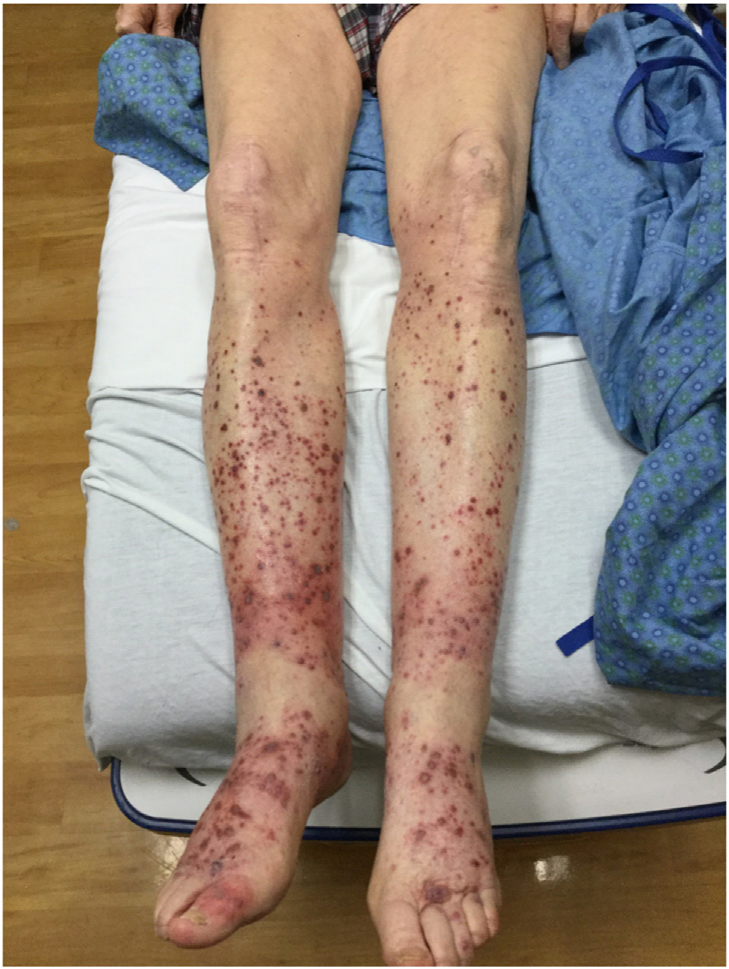
Scattered palpable purpura on the bilateral lower extremities was present on both the anterior and posterior surfaces.

A 95-year-old man with a history of myasthenia gravis on mycophenolate mofetil presented with 1 week of nonpainful, palpable purpuric papules arranged symmetrically on his lower legs. The rash was initially limited to his ankles, but new lesions developed during the next few days and progressed proximally up his legs. Notably, around 8 weeks before presentation, the patient was clinically diagnosed with zoster of a left thoracic dermatome and treated with acyclovir with no noticeable complications. On examination, scattered palpable purpura on the bilateral lower extremities was noted (Fig 1). The rash was present on both the anterior and posterior surfaces and extended from the plantar surface of the feet to the knees. On his left sixth thoracic dermatome, there was a hyperpigmented patch with overlying superficial scarring.

**Fig 2:**
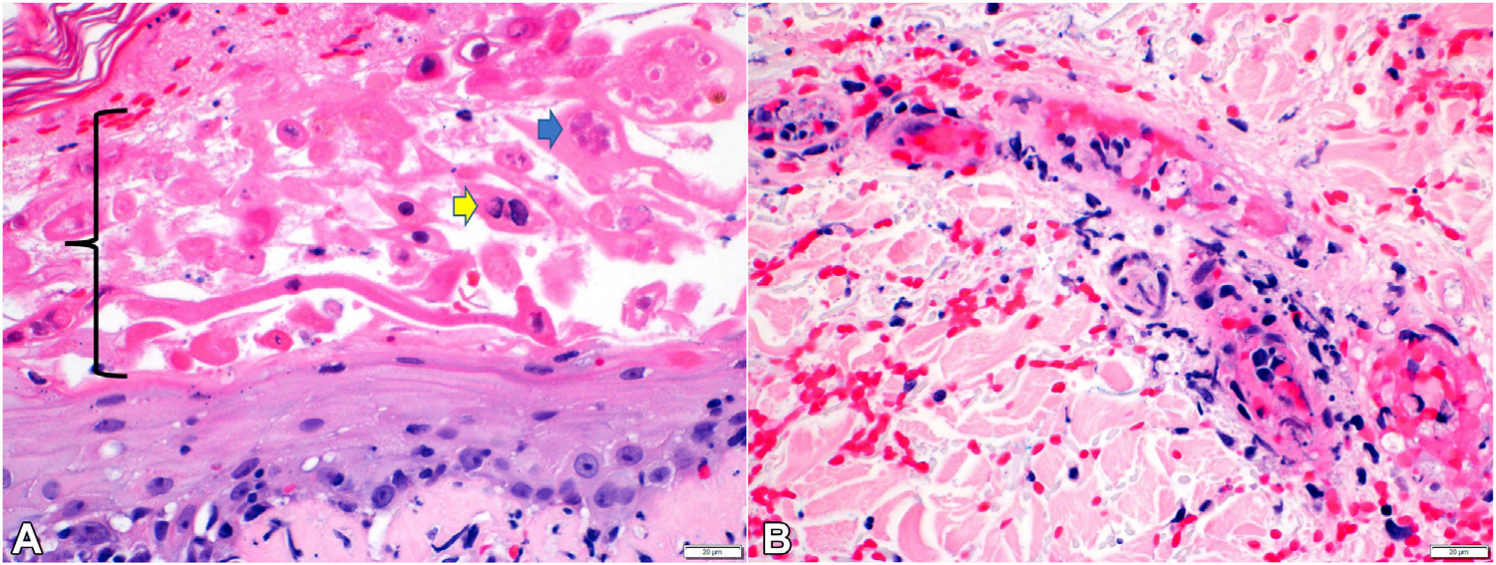
**A,** Herpetic viropathic changes (*bracketed area*) including multinucleation (*blue arrow*), chromatin margination (*yellow arrow*), and necrosis. **B,** Vascular damage immediately deep to the epidermal lesion. Hematoxylin and eosin stain. Scale bars: 20 micrometers.

**Fig 3:**
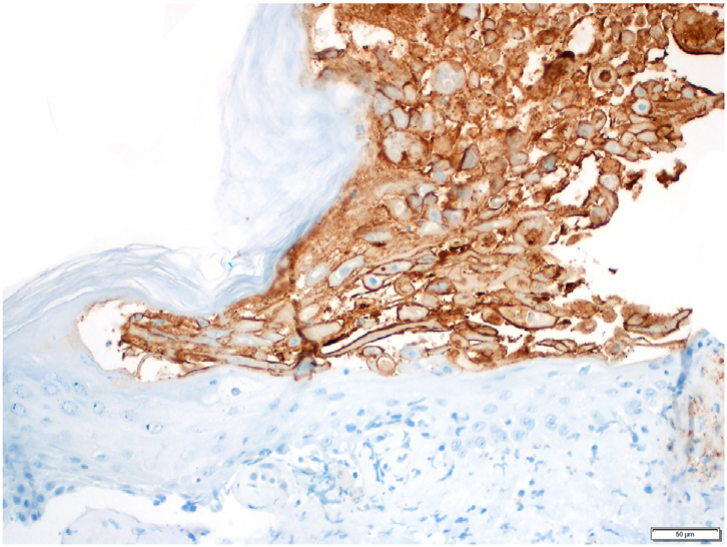
Varicella-zoster virus immunohistochemical stain, highlighting lesional keratinocytes. 3,3′-Diaminobenzidine chromogen with hematoxylin counterstain. Scale bars: 50 μM.

Histopathologic analysis of a left shin punch biopsy showed herpetic viropathic changes of the epidermis (Fig 2, *A*), with vascular damage in the underlying superficial dermis (Fig 2, *B*). VZV stain demonstrated strong labeling of lesional keratinocytes (Fig 3) with focal staining of a small dermal vessel. On immunofluorescence, there were nonspecific granular IgM, C3, and smooth fibrin deposits in the superficial vessel walls. Testing of lesional skin by polymerase chain reaction was positive for VZV. Additional workup was notable for positive antinuclear antibody but unremarkable for urinalysis, hepatitis panel, rheumatoid factor, cryoglobulins, C3, C4, antineutrophil cytoplasmic antibodies, extractable nuclear antibody panel, and serum protein electrophoresis.

Given his examination findings, the patient was initially diagnosed with leukocytoclastic vasculitis and discharged home with triamcinolone cream for symptomatic relief since his initial workup was normal. However, he was asked to return to the hospital after his biopsy and VZV polymerase chain reaction results returned, at which point he was admitted for inpatient care of disseminated zoster. Intravenous acyclovir was given for 48 hours before he was transitioned to a 21-day oral regimen. His mycophenolate mofetil was held for 2 weeks. He was discharged after 4 days with outpatient primary care and neurology follow-up.

## Discussion

VZV vasculopathy has been a well-documented phenomenon since being observed in 1896 for its association with strokes due to cerebral artery involvement.^3^ Since then, various vasculopathies have been observed secondary to VZV, such as giant cell arteritis, retinal and choroidal vasculitis, and Takayasu arteritis.^4^ There have additionally been several reports of cutaneous vasculitis related to zoster in the literature.^4^, ^5^, ^6^, ^7^, ^8^, ^9^ These presentations may represent a rare but important subset within the spectrum of VZV-related disease.

Upon literature review using the terms “zoster,” “small vessel vasculitis,” and “leukocytoclastic vasculitis,” we identified 11 similar case reports of zoster with a component of vasculitis published between 1984 and 2021. The mean patient age was 65.8 years old (range, 39-84 years old); 66.6% of patients were male and 33.3% of patients were female; 58.3% of patients were immunocompromised. Common physical examination findings included vesicles (58.3%), papules (50.0%), purpura (50.0%), ulceration (16.6%), and bullae (16.6%). Like our patient, 41.6% of previous cases showed bilateral involvement, and 58.3% of cases involved the lower limbs.^4^, ^5^, ^6^, ^7^ The unusual clinical presentation makes a diagnosis of zoster with a component of vasculitis challenging based on the history and physical examination alone. This is especially true in immunocompromised individuals, where characteristic zoster findings such as vesicles, neuropathy, pain, and pruritus may be absent.^8^

Patients who develop components of vasculitis following zoster are often initially misdiagnosed with bacterial infections, sarcoidosis, or drug reactions among other conditions.^5^^,^^7^^,^^8^ On histopathology, zoster with a component of vasculitis can closely resemble leukocytoclastic vasculitis with findings of perivascular inflammatory infiltrate, small-vessel thrombosis, and fibrin.^4^^,^^7^^,^^8^ Direct immunofluorescence can similarly show dermal vessel deposits of IgG, IgM, C3, and fibrinogen.^5^^,^^8^ Important diagnostic clues on histology are herpetic cellular changes such as acantholysis, intranuclear inclusion bodies, and/or multinucleation.^4^ At least one of these virologic sequelae was present in all previously reported cases to the best of our knowledge.

The pathogenesis of vasculitic change due to VZV has been minimally studied due to the absence of animal models and the rarity of cases.^3^ It is known that VZV infections can travel to blood vessels either transaxonally or hematogenously.^8^ Once at the vessel wall, infection can move transmurally from the adventitia to the intima and eventually involve the endothelium.^3^ Consequently, several major changes are induced, namely, a decrease in the number of smooth muscle cells in the vessel wall, disruption of the internal elastic lamina, and intimal thickening due to myofibroblast accumulation.^3^ This is accompanied during early infection by perivascular neutrophils.^3^^,^^5^ Taken together, these VZV-induced structural and inflammatory changes are thought to be the underlying mechanism behind VZV thrombogenesis and vasculitis. Notably, this mechanism is distinct from the classic immune complex deposition etiology for leukocytoclastic vasculitis.

Once a diagnosis of zoster with a component of vasculitis is made, intravenous antiviral therapy should be administered. Common agents include acyclovir, valaciclovir, famciclovir, or foscarnet for acyclovir-resistant cases.^1^ Topical antibiotics for secondary bacterial infections and analgesics for pain management may also be used as needed. Immunosuppressed patients should consider temporarily pausing their regimen.

Diagnosis of atypical presentations of VZV can be challenging, especially based on clinical features alone. Our case highlights the importance of considering this diagnosis in immunosuppressed patients with a new-onset vesicular rash on the lower extremities.

## Conflicts of interest

None disclosed.
